# Embolization of cranial dural arteriovenous fistulae with ONYX: Indications, techniques, and outcomes

**DOI:** 10.4103/0971-3026.59748

**Published:** 2010-02

**Authors:** Rashmi Saraf, Manish Shrivastava, Nishant Kumar, Uday Limaye

**Affiliations:** Division of Interventional Neuroradiology, Department of Radiology, KEM Hospital, Mumbai, India

**Keywords:** Dural arteriovenous fistula, embolization, ONYX, technique

## Abstract

**Objectives:**

The purpose of this study was to establish the role of the liquid embolic agent, ONYX, in the treatment of cranial dural arteriovenous fistulae (DAVFs) and to redefine the indications, techniques and outcomes of treatment with ONYX.

**Materials and Methods:**

This is a retrospective study of 25 DAVF patients who underwent endovascular treatment with ONYX between February 2006 and July 2008. All patients of DAVF presenting in this period were treated with ONYX.

**Results:**

Anatomic cure (i.e., complete angiographic closure of the fistula) was achieved in a single session and through a single arterial pedicle injection in 21 out of 25 patients (cure rate of 84%). Out of four patients with residual fistulae, one achieved cure that was evident on a control angiogram obtained at 3 months while three had no vascular access for further embolization and so were referred for radiosurgery. There was only one recurrence seen in angiograms obtained at the end of one year and this patient was re-embolized successfully with ONYX. Complications were seen in two patients.

**Conclusion:**

ONYX embolization of DAVFs has revolutionized the endovascular treatment of DAVFs, achieving high cure rates in a single session with minimal complications. Transarterial ONYX embolization should be the first option for all locations, except cavernous DAVFs.

## Introduction

Intracranial dural arteriovenous fistulae (DAVFs) are uncommon lesions that reportedly account for 10-15% of all intracranial arteriovenous lesions. Most of these lesions are acquired, and venous sinus thrombosis, previous surgery, ear infection, and head trauma are potential predisposing factors.[1,2] Several studies over the last two decades have shown that the clinical presentation of DAVFs is strongly related to their venous drainage pattern.[3–5] Although various classifications of DAVFs have been proposed over the years, the presence or absence of cortical venous drainage is considered to be the most important determinant for management decisions.[6–8] DAVFs that drain retrogradely into cortical veins are aggressive lesions that can present with intracranial hemorrhage, seizures, intracranial hypertension, and neuropsychiatric symptoms.[9] Other features suggestive of the aggressiveness of DAVFs are stenosis of the draining sinus, deep venous drainage, certain locations like the anterior cranial fossa and tentorium, venous congestion and venous aneurysm or varix, mainly in those DAVFs with direct leptomeningeal venous drainage.[10] Intolerable bruit and DAVFs of the cavernous sinus that present with ocular symptoms, are also indications for treatment, even without cortical venous reflux. Isolated sinuses with DAVFs are extremely aggressive lesions due to extensive cortical venous reflux. Prompt and definitive treatment is indicated for these lesions. Endovascular management is now the primary modality of treatment for all DAVFs. There have been rapid advances in endovascular techniques, hardware, and in the strategies of treatment of DAVFs. The new liquid embolic system, ONYX (ev3, CA, USA), has been found to useful in treating DAVFs. In this retrospective study, we have sought to establish the role of ONYX in the endovascular treatment of DAVFs.

## Materials and Methods

This is a retrospective study of 25 consecutive patients with DAVFs, all of whom underwent endovascular treatment with ONYX between February 2006 and July 2008. The treatment criteria included aggressive symptoms (hemorrhage, neurological deficit, cortical venous reflux, direct leptomeningeal venous drainage) and intolerable benign symptoms like bruit or severe headaches. Table 1 represents the clinical presentations of the DAVFs as per their locations. DAVFs of the cavernous sinus, mainly of the anterior compartment with good transarterial access, were also treated with ONYX embolization. There were 23 males and two females whose age ranged from 12 to 68 years. The locations of the fistulae in these 25 patients were: Transverse sigmoid (9), all left sided, tentorial (3), anterior cranial fossa (2), cavernous sinus (2), foramen magnum (3), sphenoid (1), petrosal (1), sigmoid sinus (1), middle cranial fossa (2), and frontal intraosseous (1). Three patients had a prior history of venous sinus thrombosis and one patient reported a history of head trauma in the past while one was incidentally diagnosed when evaluated for ischemic stroke.

**Table 1:** Dural AVFs

Patient no.	Location	Headache	Seizures	Tinnitus	Ocular	Progressive neurological deficit	Papilloedema	ICH	SAH	Memory loss
1	Sphenoid	+	−	−	−	−		+	−	−
2	Tentorial	+	−	−	−	−	+	−	−	+
3	Transverse sinus	+	−	+	−	−		−	−	−
4	Transverse sinus	−	−	+	−	−		−	−	−
5	Middle cranial fossa	−	−	−	+	−	+	−	−	−
6	Transverse sinus	+	−	−	−					
7	Transverse sinus	+	−	−	−	−		+	−	+
8	Foremen-magnum	+	−	−	−	−		−	+	−
9	Transverse sinus	+	+	−	−	+		+	−	+
10	Frontal intraosseous	−	−	−	+	−		−	−	−
11	Sigmoid	+	−	+	−	−		−	−	−
12	Tentorial	+	−	−	−	−		+	−	−
13	Cavernous	+	−	−	+	−		−	−	−
14	Transverse-sigmoid	+	−	−	−	−	+	−	−	−
15	Middle cranial fossa	−	−	−	−	−		−	−	−
16	Transverse sinus	+	−	−	−	−		+	−	−
17	Anterior cranial fossa	+	+	−	−	−		+	−	−
18	Transverse sinus	+	−	−	−	−	+	+	−	−
19	Tentorial	+	+	−	−	−		−	−	−
20	Transverse sinus	+	+	−	−	−		+	−	−
21	Transverse sinus	+	+	−	−	+		−	−	−
22	A-terior cra-ial fossa	+	−	−	+	−		+	−	−
23	Foremen-magnum		−	−	−	+		−	−	−
24	Foremen-magnum	+	−	−	−	−		−	+	−
25	Petrosal	+	−	−	−	−		−	+	−

Cortical venous reflux was seen in 17 patients and direct leptomeningeal venous drainage was seen in four patients. One patient had a middle cranial fossa DAVF draining into the superior ophthalmic vein, leading to ocular symptoms. Three patients had an osteodural type of DAVF. One patient with a foramen magnum DAVF showed reflux into the perimedullary venous system. All patients with foramen magnum and petrosal DAVFs showed direct drainage into the leptomeningeal veins with venous pouches, which then refluxed into multiple cortical veins. Four out of nine patients with transverse sigmoid DAVFs had an isolated sinus. One transverse sigmoid DAVF patient had been embolized previously with transvenous coiling and one patient with an osteodural sphenoid wing DAVF had undergone two sessions of transarterial glue embolization previously.

### Clinical presentation

**Figure 1 (a-f):**
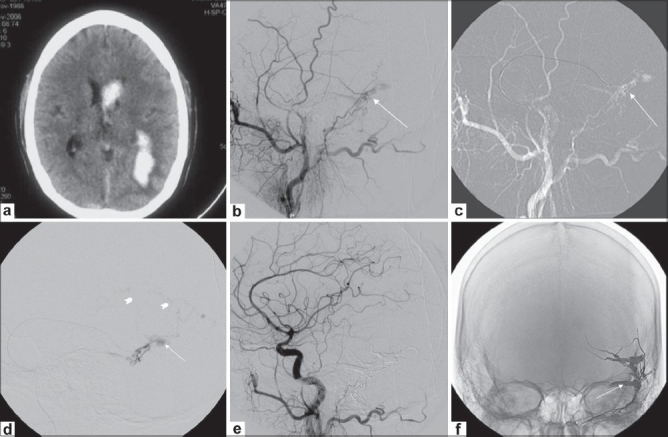
Left petrosal dural arteriovenous fistula (AVF). CT scan of the brain (a) shows left parieto-occipital hemorrhage with intraventricular extension Left external carotid angiogram (b) shows a left petrosal dural AVF (arrow). Left external carotid angiogram (c) with a road map image shows the final position of the microcatheter (arrow) in the middle meningeal artery. Microcatheter angiogram (d) shows the isolated petrosal sinus (arrow) with extensive cortical venous reflux (arrowheads). 2 mL of Onyx 18 was injected from this site over 40 min. Postembolization left common carotid angiogram (e) shows exclusion of the fistula. Plain skull radiograph (f) shows the Onyx cast (arrow)

**Figure 2 (a,b):**
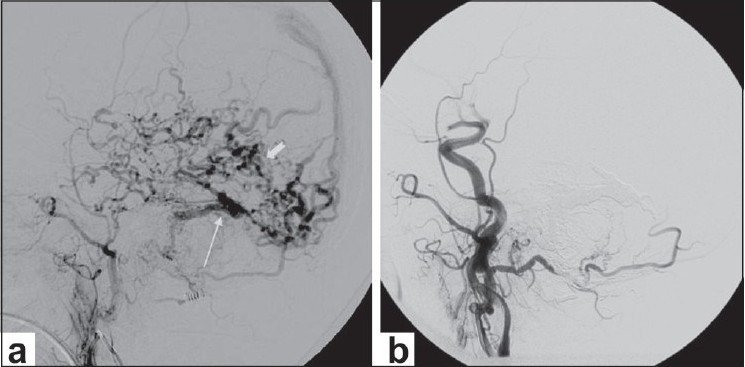
Isolated sigmoid dural arteriovenous fistula (AVF). Left external carotid angiogram (a) shows an isolated sigmoid dural AVF (arrow) with extensive cortical venous reflux (arrowhead). Postembolization left common carotid angiogram (b) shows angiographic closure of the fistula

Intracranial hemorrhage was the presenting symptom in 12 out of 25 patients (48%). Nine out of these 12 had intracerebral hemorrhage (ICH), with subarachnoid hemorrhage (SAH) in three patients. Among the ICH patients, transverse sigmoid sinus was the most frequent location followed by tentorial, anterior cranial fossa, petrosal [Figure 1], and osteodural sphenoid wing DAVFs. SAH was the predominant manifestation in foramen magnum DAVFs. Other presentations were headaches (76%), seizures (20%), tinnitus (16%), ocular symptoms (16%), neuropsychiatric symptoms (8%), and progressive neurodeficit (8%). Two patients had neuropsychiatric symptoms and both showed extensive venous congestion [Figure 2] on angiography, with a fistula in the left transverse-sigmoid location. One had features of chronic venous congestion on CT scan. One of these patients had memory disturbances and the other with chronic venous congestion had progressed to altered sensorium. One patient with a tentorial DAVF presented with progressive decrease in vision. Ocular symptoms were seen in patients with cavernous sinus DAVFs, the middle cranial fossa DAVF draining into the superior ophthalmic vein (SOV) and the right frontal osteodural AVF draining into a venous pouch and then into the SOV.

Four patients had papilledema, two of whom presented with blurring of vision; all of them had transverse-sigmoid DAVFs. Four out of nine patients with transverse-sigmoid DAVFs and one patient with an anterior superior sagittal sinus DAVF had an isolated sinus, and all presented with ICH. Eight out of nine patients with transverse-sigmoid DAVFs had thrombosis of the sigmoid sinus evident on MRI or angiography. One case with torcula and medial transverse sinus DAVF had no cortical venous reflux, although intracranial hypertension was present as evidenced by bilateral papilledema. One patient with a sigmoid DAVF presented with intolerable bruit although there was no cortical venous reflux (CVR).

### Treatment strategy

Endovascular treatment is the primary modality of treatment of all DAVFs at our center for complete cure. The liquid embolic agent, ONYX (ethylene vinyl alcohol copolymer, ev3), was used for treatment in all the patients. ONYX is available in two concentrations ONYX 34 (more viscous) and ONYX 18. Six-vessel angiography was performed in all patients to study the angioarchitecture of the shunt precisely and to plan the treatment.

### Endovascular procedure/technique

**Figure 3 (a-d):**
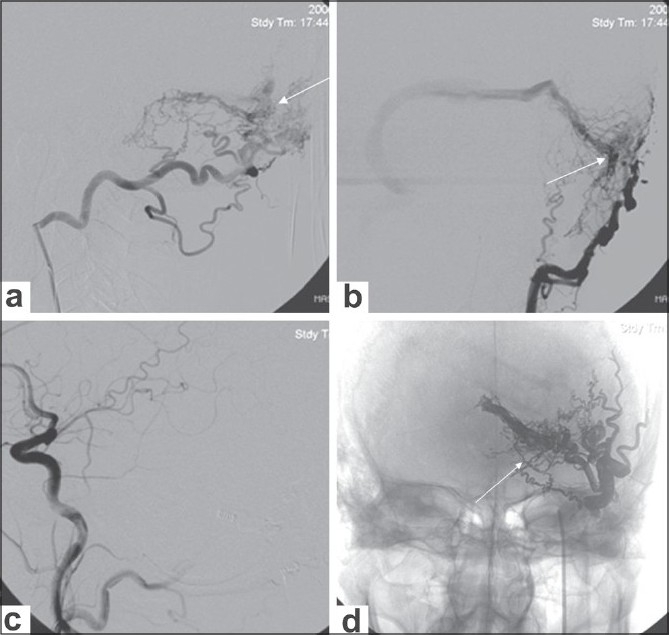
Left transverse dural arteriovenous fistula (AVF). Lateral (a) and oblique (b) left occipital angiograms show a left transverse dural AVF (arrow). Transarterial embolisation was performed through the occipital artery with ONYX. Postembolization left common carotid angiogram (c) shows occlusion of the fistula. Plain radiograph (d) shows the ONYX cast (arrows)

Angiography and embolization were performed on a biplane angiographic unit (Integris Allura, Philips Medical Systems). Treatment was done under general anesthesia and systemic heparinization. A 5F guiding catheter was used for the external carotid artery (ECA) and 6F for the internal carotid artery (ICA) and vertebral artery (VA). Dimethyl sulfoxide (DMSO) compatible microcatheters (Ultraflow, Marathon, Echelon, Rebar,ev3) were used for superselective cannulation of the predominant feeding arteries. Microguidewires used were Mirage or X-Pedion (ev3). The microcatheter was positioned at the fistula site as close to the draining sinus or vein as possible. A microcatheter angiogram was taken to confirm the position of the catheter and to see the drainage of the fistula. The microcatheter was flushed with 10 mL of normal saline and the dead space was flushed with 0.25 mL of DMSO injected slowly over 40 sec. The ONYX vial was shaken on the ONYX mixer for at least 20 min prior to its use. The ONYX injection was then started slowly-initially 0.2 mL without guidance and subsequently under biplane roadmap guidance. Injection was stopped for a few minutes when the initial reflux on the microcatheter was seen to form a plug. Whenever reflux occurred, injection was stopped for sometime. Patience during injection is the key to achieve cure with ONYX. Embolization was performed until angiographic cure was achieved [Figure 3]. The microcatheter was withdrawn rapidly at the end of each procedure. Postoperative anticoagulation was done only in patients with marked stasis or slow flow in cortical veins.

During embolization through the ascending pharyngeal artery, care was taken to remain as close to the fistula as possible to minimize ischemic injury to the cranial nerves. Embolization through transosseous branches of the occipital artery required a lot of patience and a large volume of ONYX as ONYX penetration starts only after 25-30 minutes.

Protective balloons should be used in cases with high-flow DAVFs with large supplies from the vertebal or the internal carotid arteries. Flow control can be achieved with manual compression of the carotids or both the jugular vein and the carotid, or by balloon occlusion of the venous sinus. In high-flow DAVFs, the injection should be started with ONYX 34 and, if needed, continued with ONYX 18. Reflux on the microcatheter is not much of a problem in embolization through the ECA branches.

## Results

The results of the study have been recorded in terms of the location, clinical presentation, angiographic outcome and complications [Table 1]. Also the volume of ONYX injected and the duration of injections were recorded. All embolizations were transarterial.

**Figure 4 (a-e):**
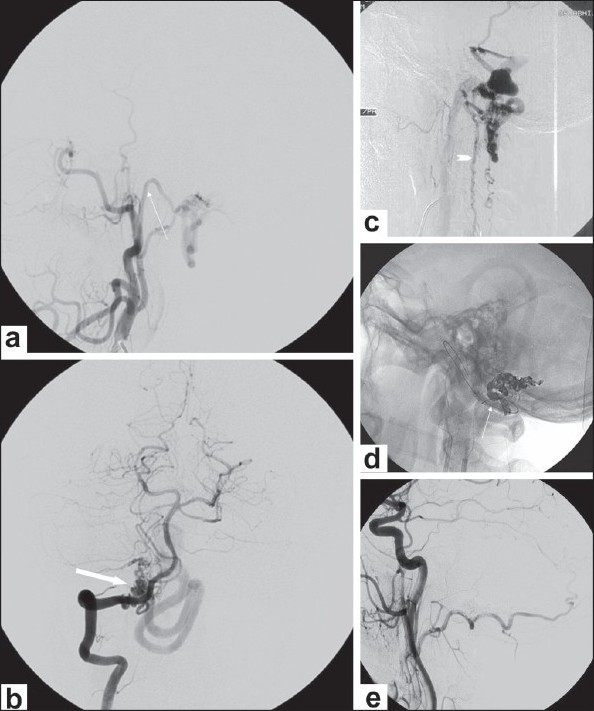
Foramen magnum dural arteriovenous fistula (AVF). Right external carotid (ECA) (a) and right vertebral (b) angiograms show a foramen magnum dural arteriovenous fistula which is supplied by branches from the ascending pharyngeal (arrow) and posterior meningeal (thick arrow) arteries. Right ECA angiogram shows reflux into the spinal perimedullary venous system (arrowhead in (c). Embolization was performed through the ascending pharyngeal artery with balloon placement in the right vertebral artery arrow in (d). Post-embolization right ECA angiogram (e) shows obliteration of the fistula

In 21 out of 25 patients, angiographic cure (*i.e*., complete closure of the fistula) was achieved in a single session and through a single arterial pedicle injection. In only one patient with a high-flow torcula and a medial transverse sinus DAVF with multiple feeders, were two sessions performed and four arteries were embolized. A slow-flow residue was seen at the end of the last session and also in the one-year follow-up angiography. The remaining three who had slow-flow residual fistulae did not have access for further embolization and hence, were referred for radiosurgery. Flow control was achieved in one patient with a left transverse-sigmoid DAVF by balloon placement in the transverse sinus. In two patients with high-flow DAVFs, balloons were placed in the internal carotid artery and the vertebral artery [Figure 4] respectively, to prevent the reflux of ONYX into these arteries that were supplying the fistulae through their meningeal branches. In both the patients of isolated TS DAVFs, cure was achieved through a single pedicle injection.

A total of 28 arteries were embolized: The middle meningeal artery in 17, the occipital artery in four, distal branches of the internal maxillary artery in three, the ascending pharyngeal in two, the ophthalmic artery in one, and the dural branch of the VA in one. Embolization of only one feeder was performed in 24 out of 25 patients. The volume of ONYX injected ranged from 0.4 to 5 mL per procedure. The duration of injection per pedicle ranged from 15 to 90 min. The duration of injection was longer in transosseous branches of the occipital artery and the volume of ONYX used was also higher. In high-flow situations, the initial injection was done with ONYX 34 (volume ranging from 0.5 to 1.5 mL) and then continued with ONYX 18. In one patient with a high-flow cavernous DAVF, one coil was placed initially and then embolization was performed using ONYX 34.

All patients were extubated postoperatively and careful observation was done in the Intensive Care Unit for 24 h. Only one patient was electively ventilated for 24 h as there was marked stasis in the congested veins after closure of the fistula. Anticoagulation was done for 48 h. Patients were followed up at one month and at three and six months with cerebral angiography and MRI respectively. Control angiography was performed six months post procedure in 20 patients of whom 16 showed complete cure; a control angiogram is awaited in five patients. Clinically, all the patients had resolution of symptoms after the procedure and in the follow-up period (including those with residual fistulae).

The cure rate for DAVFs with ONYX embolization was 84% with stable occlusion of the fistula as seen on control angiograms.

### Complications

Two patients had complications after treatment: In one patient, a venous pouch was present at the cerebellopontine angle which thrombosed after fistula closure, causing a mass effect. Surgical decompression was done and the postoperative course was uneventful. One patient with a cavernous dural fistula developed a left parietal hematoma within 24 hours of the procedure. However, good clinical recovery was observed. Only one patient with a high-flow TS DAVF with features of chronic venous congestion prior to the procedure, had long-term morbidity and a poor outcome.

One patient with a right frontal intraosseous DAVF (a case of CAMS II) had ICH one year later due to the temporal AVM; no residual fistula was seen.

## Discussion

Dural AVFs are uncommon intracranial lesions consisting of direct, abnormal connections between the meningeal arteries and the dural venous sinuses or leptomeningeal veins. Their symptoms are highly variable. Various studies have shown a strong correlation between the associated aggressive symptoms and the presence of cortical venous reflux. The aim of the treatment should be complete closure of the shunt by occluding the proximal vein or the diseased sinus if it is not taking part in the drainage of the brain.

The treatment options available are surgery, radiosurgery,[11] and embolization.[12] There have been technical advances in all the fields with improving cure rates over the last two decades. A multidisciplinary approach was used initially for complex DAVFs.[13,14] Surgery and radiosurgery have shown mixed results.[15,16] The results of embolization have improved over time due to rapid advancements in endovascular techniques (highly flexible hydrophilic catheters, wires, and advanced embolic agents). Mullan *et al*.[17] first described embolization of a DAVF of the cavernous sinus in 1979. Hallbach *et al*.[18] (1987) first reported transvenous embolization of a transverse-sigmoid DAVF. With advances in microcatheters, microwires, and coils, transvenous embolization became the treatment option for these locations.[19,20] Transarterial embolization was performed with polyvinyl alcohol (PVA) particles to resolve symptoms or in combination with other procedures such as irradiation, surgery, or transvenous embolization. Complex dural fistulae were still difficult to treat, especially those in which the sinuses were thrombosed distally as well as proximal to the fistulous segment, also known as an isolated sinus. Transosseous direct puncture and packing of the isolated sinus have been established as primary treatment options in isolated sinuses with DAVFs.[21,22] In DAVFs with direct leptomeningeal venous drainage and in some cases of cavernous DAVF (mainly the anterior compartment ones), treatment with dilute *N*-Butyl cyanoacrylate (NBCA) has shown good results.[23] The drawbacks of NBCA[35] use are the need for a highly experienced operator, appropriate glue concentrations, and unpredictable outcomes (too proximal or too distal embolisation lead to hazardous complication or incomplete fistula closure). Recent studies have reported the use of ONYX by the transvenous route for cavernous dural fistulae.[24] Surgery and radiosurgery are used these days only in those patients with endovascular treatment failure.

**Figure 5 (a-f):**
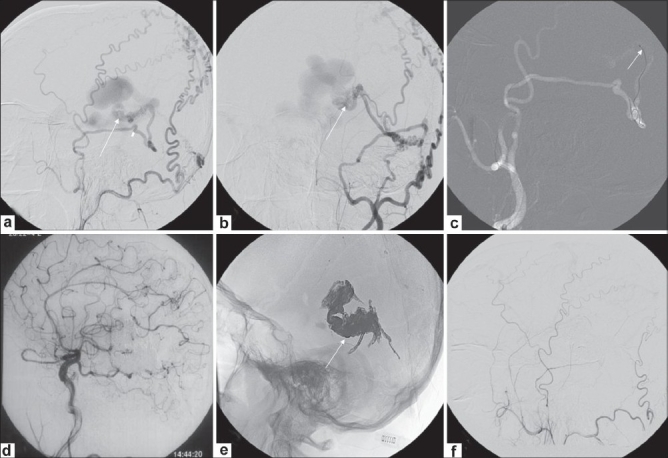
Falco-tentorial dural fistula. Frontal (a) and lateral (b) left external carotid angiograms show a high flow falco-tentorial dural fistula (arrow) supplied by the middle meningeal artery (arrowhead). Road map image (c) shows the position of the microcatheter (arrow) in the distal middle meningeal artery close to the venous pouch. Postembolization left common carotid angiogram (d) shows complete closure of the fistula. The Onyx cast arrow in (e) is seen with penetration into the venous pouch. Lateral left ECA angiogram (f) obtained after six months shows no evidence of the fistula

The advent of a new liquid embolic system, ONYX (ev3), with its unique physical properties has revolutionized endovascular treatment of intracranial DAVFs. ONYX is a nonadhesive liquid embolic agent comprising of an ethylene vinylalcohol copolymer dissolved in DMSO with tantalum powder for radio-opacity. It allows prolonged injections, larger volumes of injections, and greater variation in the rate of injection from the single pedicle.[25–28] The reflux-hold-reinjection technique allows highly predictable and thorough penetration, especially of multiple microfistulae along the wall of the sinus supplied by multiple feeders. The excellent endovascular handling characteristics of ONYX allow complete occlusion of the diseased sinus or proximal vein without reflux into the cortical veins, thus minimizing complications [Figure 5]. The injection can be interrupted and guiding catheter contrast injections can be done to assess the completion of the procedure, which is not possible with NBCA. Two concentrations of ONYX are available at our center, ONYX 18 and ONYX 34. The lower the concentration of the polymer, the more is the distal penetration achieved. Placing the ONYX vial on an ONYX heater for 5-10 min at 70°C before injection, may further enhance penetration and flow of ONYX. ONYX is a permanent agent that has not been found to cause much inflammation in animal studies, hence, long-term or permanent cure is expected.[29,30] In this study of 25 patients, 17 had cortical venous reflux and four had direct leptomeningeal venous drainage. The remaining four had intolerable bruit or ocular symptoms. Intracranial hemorrhage was the predominant presenting symptom. In 21 out of 25 cases, complete closure of the fistula by ONYX embolization was achieved in a single injection from a single feeder, thus achieving a cure rate of 84%.

The maximum duration of injection was 90 min with easy retrievability of microcatheters at the end of the injection in all cases Two cases of isolated sinus were effectively treated with ONYX embolization in a single session, thus obviating the need for direct puncture and packing of sinuses which is cumbersome and time consuming.

Postprocedure complications were noted only in two patients, of which one patient with a cavernous DAVF had a small parietal hematoma distant from the fistula site, and the other had thrombosis of the venous pouch which was successfully managed with surgical intervention. Good outcome is not expected in patients presenting with chronic venous congestion. In patients with extensive cortical venous reflux and venous congestion of the brain, the closure of the fistula increases the propensity of thrombosis of these arterialized cortical veins. These patients should be managed with low molecular weight heparin for at least 48 hours after the procedure.

Various case reports in the last two years have described the efficacy of transarterial ONYX embolization in DAVFs of the anterior cranial fossa, superior sagittal sinus, lesser sphenoid wing, clival DAVFs, and in the isolated sinus.[31–34] In their study of six DAVF patients who were treated with ONYX, Tulgoat *et al*.[35] stated that ONYX 18 is a safe treatment and when performed in optimal conditions, fills the total DAVF and its drainage vein or sinus after a single arterial feeder catheterization. Nogueira *et al*.[36] performed a retrospective analysis of 12 consecutive patients with intracranial DAVFs who were treated with ONYX as the single treatment technique. Cure was noted in ten out of 12 patients; there was no significant morbidity or mortality. They concluded that endovascular treatment of intracranial DAVFs with ONYX is feasible, safe, and highly effective with a small recurrence rate in the short-term follow-up. Carlson *et al*.[37] reported a series of six patients with symptomatic DAVFs who were treated initially with transarterial ONYX embolization and other endovascular techniques and achieved cure in five patients. None of the patients had worsening of neurological function. They concluded that transarterial ONYX embolization and other endovascular methods can angiographically obliterate DAVF and even allow occlusion of multiple arterial feeding arteries from a single arterial injection. Cognard *et al*.[38] performed a study of 30 patients of DAVF with cortical venous reflux (CVR), who had been treated with ONYX embolization. Cure rate after embolization with ONYX was 80% (24 out of 30 patients) and complications occurred in two patients.

## Conclusion

This study emphasizes the role of ONYX in the treatment of intracranial DAVFs. ONYX has unique physical properties, which facilitates prolonged injections that can be better controlled, and have a more predictable penetration. Transarterial ONYX embolization should be the first option for all locations, except cavernous DAVFs. ONYX embolization of an isolated sinus with DAVF should replace direct puncture and packing of sinuses. To conclude, ONYX embolization has revolutionized the endovascular treatment of DAVFs, achieving higher cure rates in single sessions with minimal complications. With our experience of ONYX, we recommend ONYX as the primary treatment modality in DAVFs of all locations, except cavernous DAVFs.
